# Bacterial expression of human kynurenine 3-monooxygenase: Solubility, activity, purification^☆^

**DOI:** 10.1016/j.pep.2013.11.015

**Published:** 2014-03

**Authors:** K. Wilson, D.J. Mole, M. Binnie, N.Z.M. Homer, X. Zheng, B.A. Yard, J.P. Iredale, M. Auer, S.P. Webster

**Affiliations:** a Drug Discovery Core, University/BHF Centre for Cardiovascular Science, Queen's Medical Research Institute, The University of Edinburgh, 47 Little France Crescent, Edinburgh EH16 4TJ, United Kingdom; b MRC Centre for Inflammation Research, Queen's Medical Research Institute, The University of Edinburgh, 47 Little France Crescent, Edinburgh EH16 4TJ, United Kingdom; c Mass Spectrometry Core, University/BHF Centre for Cardiovascular Science, Queen's Medical Research Institute, The University of Edinburgh, 47 Little France Crescent, Edinburgh EH16 4TJ, United Kingdom; d School of Biological Sciences and School of Biomedical Sciences, University of Edinburgh, CH Waddington Building, The University of Edinburgh Kings Buildings, Mayfield Road, Edinburgh EH9 3JD, United Kingdom

**Keywords:** 3-Hydroxykynurenine, Kynurenine 3-monooxygenase, Purification, Solubility

## Abstract

Kynurenine 3-monooxygenase (KMO) is an enzyme central to the kynurenine pathway of tryptophan metabolism. KMO has been implicated as a therapeutic target in several disease states, including Huntington’s disease. Recombinant human KMO protein production is challenging due to the presence of transmembrane domains, which localise KMO to the outer mitochondrial membrane and render KMO insoluble in many *in vitro* expression systems. Efficient bacterial expression of human KMO would accelerate drug development of KMO inhibitors but until now this has not been achieved. Here we report the first successful bacterial (*Escherichia coli*) expression of active FLAG™-tagged human KMO enzyme expressed in the soluble fraction and progress towards its purification.

## Highlights

• This is the first report of soluble and active bacterially expressed human KMO protein.
• Partial purification of the enzyme was achieved and the two protein co-elutants identified.
• Steady state kinetic parameters were comparable to those reported for mammalian expressed.
• The C-terminal membrane targetting domain of human KMO is required for its enzymatic activity.

## Introduction

In mammals, the kynurenine pathway is the route of 99% of dietary tryptophan metabolism and is the primary route of synthesis of the essential cellular cofactor nicotinamide adenine dinucleotide (NAD)^1^ [1,2]. Kynurenine metabolism produces several biologically-active intermediates with diverse physiological actions which participate in the pathogenesis of various neurodegenerative disorders, including Alzheimer’s disease, Parkinson’s disease, Huntington’s disease [3] and sterile systemic inflammation [4,5]. The kynurenine pathway has an established mechanistic role in immune regulation [6].

**Fig. 1:**
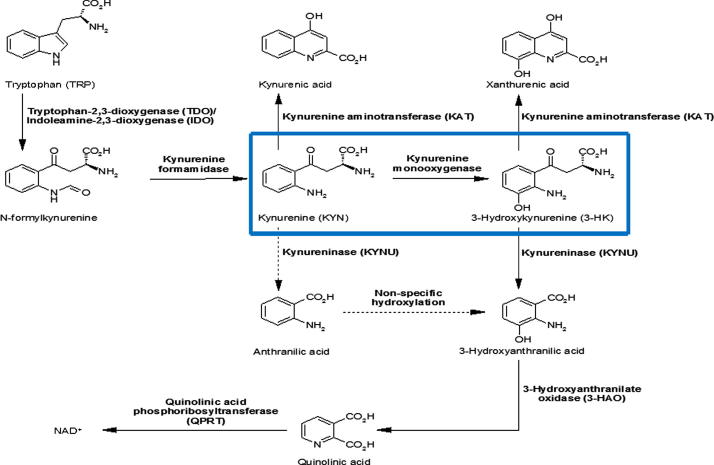
Kynurenine pathway. Biochemical pathway of tryptophan breakdown, KMO highlighted by blue box (adapted from Crozier-Reabe, 2008 [10]). (For interpretation of the references to colour in this figure legend, the reader is referred to the web version of this article.)

Central to the kynurenine pathway is the NADPH-dependant flavoprotein hydroxylase, kynurenine 3-monooxygenase (KMO) (EC 1.14.13.9) (Fig. 1). This enzyme catalyses the specific hydroxylation of kynurenine (L-Kyn) at the 3 position of its phenol ring to generate 3-hydroxykynurenine (3-HK) [3]. KMO protein is monomeric and has a molecular mass of 50 kDa with 1 mol of non-covalently bound flavin adenine dinucleotide (FAD) per mol of protein monomer [7]. FAD is very tightly bound to KMO and is slow to dissociate [8]. The protein has been shown to undergo post-translational modification when expressed in mammalian cells and three N-glycosylation sites have been identified [8]. KMO is contained within macrophage and microglial cells, with low KMO levels distributed throughout all regions of the brain and high levels found in the liver and kidney [3]. The enzyme is believed to be localised to the outer mitochondrial membrane due to the presence of a proposed transmembrane domain near its C-terminus [9]. 3-HK, the product of KMO catalysis, exhibits toxicity to cells through reactive oxygen species generation, the cross-linking of proteins and mitochondrial respiratory chain inhibition [10]. KMO has recently been implicated as a therapeutic target for both Huntington’s disease [11] and post-traumatic sepsis [4].

Currently, there are no successful reports of bacterially expressed human KMO protein in the scientific literature and consequently no crystal structure is available for the human enzyme. Expression of active human enzyme has previously been reported in a mammalian expression system and demonstrated a *K_m_* of 153 and 100 μM for substrates NADPH and kynurenine respectively [8]. Purification of the rat (*Rattus norvegicus*) [12] and bacterial (*Pseudomonas fluorescens*) [13] enzymes has been demonstrated. Rat KMO has high sequence homology with human KMO, but *P*. *fluorescens* KMO exhibits low sequence homology when compared with the human enzyme. More recently expression of human KMO in insect cells has been reported, however, problems due to low yields and poor stability were described and the purity of the protein was not indicated or the enzyme characterised [14]. The crystal structure of *Saccharomyces cerevisiae* KMO has recently been reported [14]. It is believed that the active site residues identified in the yeast KMO crystal structure are virtually identical in human KMO [14]. However, human KMO has a region of hydrophobic amino acids at the C-terminus, believed to be the mitochondrial membrane anchoring domain, which is required for enzymatic activity and is also thought to contribute to the low aqueous solubility of the protein [8]. It should be noted that the protein construct used to obtain the yeast KMO crystal structure was a truncated version of the enzyme which did not contain the equivalent membrane targeting domain [14]. Importantly, targetted truncation experiments on the pig KMO enzyme show that the hydrophobic C-terminus has a dual role, participating in membrane anchoring and enzymatic activity [15]. It is recognised that pig KMO appears to form protein-protein complexes with two other outer mitochondrial membrane proteins: cytochrome b5 and monoamine oxidase subunit B. Uemera et al [7] found removal of these contaminants without losing enzymatic activity to be challenging, suggesting that KMO requires a membranous environment for efficient folding and function.

The FLAG™-tag is a polypeptide (DYKDDDDK) with several uses in recombinant protein production. The epitope can be utilised for isolation of fusion proteins by affinity chromatography and specific recognition by commercially available antibodies [16]. Because the FLAG peptide tag is hydrophilic, it is exposed and accessible on the surface of the fusion protein, and the fusion partner portion is less likely to be functionally altered [16]. FLAG tags can be multiplexed to enhance epitope specificity, for example, the 3×FLAG tag contains three consecutive FLAG sequences.

Our aim in this work was to express functional human KMO in a bacterial expression system and to optimise solubility and purification with the use of cleavable fusion protein tags.

## Materials and methods

### Gene synthesis and cloning

Codon-optimised synthetic genes were prepared by Genscript and provided in the vector pUC57.

#### Truncated KMO (trKMO)

**Fig. 2:**
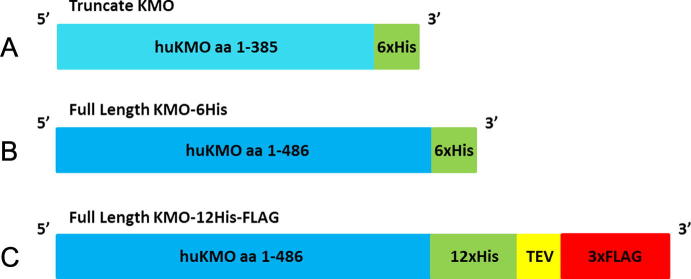
Protein construct drawings. Cartoon illustrating the composition of each protein construct, (A) truncated hKMO-6 His; (B) full-length hKMO-6×His; (C) full length hKMO-12His-3×FLAG.

A codon-optimised synthetic gene consisting of the codons for amino acids 1-385 of human KMO was prepared as above with an NdeI restriction site incorporated at the 5′-end and a NotI restriction site at the 3′-end. The gene was removed from vector pUC57 by restriction digest with NdeI and NotI and ligated into the vector pET24b (Novagen) for expression in *Escherichia coli* (Fig. 2A). The truncated gene was customised without a stop codon to allow incorporation of a C-terminal 6× polyhistidine tag contained within the pET24b vector.

#### Full length KMO-6His (flKMO)

The full length gene (GenBank Accession No. NM_003679) (Arg 452 variant) was prepared as above with an NdeI restriction site at the 5′-end and a NotI restriction site at the 3′-end (the alternative Cys 452 polymorphic variant was not prepared). The gene was removed from vector pUC57 by restriction digest with NdeI and NotI and ligated into the vector pET24b (Novagen) for expression in *E*. *coli* (Fig. 2B). The full length gene was customised without a stop codon to allow incorporation of a C-terminal 6× polyhistidine tag contained within the pET24b vector.

#### Full length KMO-12His-FLAG (KMO-FLAG)

For expression as a FLAG tagged gene, the full length gene (Arg 452 variant) was designed with sequences encoding a 12× poly histidine tag (CATCATCACCATCACCATCATCATCACCATCACCAT), TEV protease site (GAAAACCTGTATTTTCAGGGT) and 3×FLAG epitope (ATGGACTACAAAGACCATGACGGTGATTATAAAGATCATGATATCGATTACAAGGATGACGATGACAAGTGA) at the 3′-end of the KMO gene. The gene incorporated a NheI restriction site at the 5′-end and a NotI restriction site after the stop codon at the 3′-end. The gene was removed from pUC57 and ligated into the vector pET24b (Fig. 2C.).

### Bacterial cell expression

#### Truncated KMO

Truncated KMO was expressed in the *E*. *coli* cell strain BL21(DE3) pLysS (Invitrogen, C606010). Bacterial cells transformed with the pET24b-trKMO plasmid were grown in LB broth with antibiotics kanamycin (Sigma Aldrich, 60615) and chloramphenicol (Sigma Aldrich, C0378) at 37 °C with shaking at 250 rpm until the optical density at 600 nm was 0.8. Cells were then induced with 1 mM Isopropyl β-D-1-thiogalactopyranoside (IPTG) (Sigma Aldrich, I5502) for 4 h at 37 °C. Bacterial cells were collected by centrifugation at 6000 rpm for 10 min at 4 °C. Cell pellets were frozen at −20 °C until preparation of cell free extract.

#### Full length KMO-6His (flKMO) and KMO-12His-FLAG

Full length and KMO-FLAG were expressed as above with induction at 18 °C for 16 h. Bacterial cells were collected by centrifugation at 6000 rpm for 10 min at 4 °C. Cell pellets were frozen at −20 °C until preparation of lysate.

### Validation of expression

Lysed bacterial cell expression samples were examined by SDS-PAGE with simplyblue (Invitrogen, LC6060) staining and analysed by Western blot. Anti-His(C-term)-HRP antibody (Invitrogen, R931-25) (1:2000 dilution) was used to detect the truncate and flKMO proteins, and anti-FLAG M2 antibody (Sigma Aldrich, F1804) (1:1000) with goat anti-mouse HRP conjugate secondary antibody (Abcam, ab6832) (1:10 000) was used to detect KMO-FLAG protein.

### Activity assay

Standard protein assay procedure using bio-rad reagent (Bio-Rad, 500-0006) and BSA protein standards (Sigma Aldrich, P0914-10AMP) were used to determine the total protein concentration in the cell free extract. Cell free extracts were analysed for KMO enzymatic activity by measuring the conversion of l-Kyn to 3-HK detected by liquid chromatography–mass spectrometry (LC–MS/MS) in a protocol similar to that reported by Courtney et al [17]. 200 μg Total protein was incubated in 4 mM MgCl_2_ (Melford, M0533), 1 mM NADPH (Sigma Aldrich, N5130) and 200 μM l-Kynurenine (Sigma Aldrich, K8625) in 20 mM HEPES, pH7 for 2 h at 37 °C with gentle shaking at 250 rpm in a total assay volume of 100 μl. Assay samples were added to 500 μl acetonitrile (VWR, 200.60.320) with 25 μg/ml d5 Trp (Sigma Aldrich, T9753) (internal standard) to terminate activity and centrifuged at 4000 rpm for 20 min to pellet the precipitate. The supernatant was removed and dried under nitrogen and the residue re-suspended in 30:70 methanol:water with 0.1% formic acid for LC–MS/MS analysis.

For the determination of steady state kinetic parameters for the substrate l-Kynurenine, NADPH was added at a concentration of 2 mM with glucose-6 phosphate (Sigma Aldrich, G7879) (2 mM) and glucose-6 phosphate dehydrogenase (Sigma Aldrich, G6378) (1 unit per assay) to maintain the NADPH concentration. l-Kynurenine was added at concentrations of 2, 1, 0.5, 0.25, 0.125, 0.062, 0.031, 0.015 mM. For co-factor NADPH dependence of the enzymatic activity, l-Kynurenine was added in excess at a concentration of 500 μM. NADPH was added at a concentration of 100, 50, 25, 12.5, 6.2, 3.1, 1.5, 0.75 μM. In each case assays were performed in duplicate.

LC–MS analysis was carried out using the TSQ Quantum Discovery triple quadrupole mass spectrometer (Thermo Fisher Scientific, Hemel Hempstead, UK). The column used was a pentafluorophenyl (PFP) fused pore column, the column temperature was set at 40 °C. The injection volume was 10 μl and the flow rate was 500 μl/min. The method had a run time of 4 min and d5 Tryptophan was used as an internal standard. Qualifier and quantifier peaks were identified for 3-HK and for d5 Tryptophan. Data was acquired and processed using Xcalibur 1.4 and LC Quan 2.0 SP1 software packages.

### Membrane protein isolation protocol

#### Full length KMO-6His

Bacterial cell pellet containing expressed flKMO was thawed and re-suspended in 20 mM HEPES pH 7, 1 mM DTT (Sigma Aldrich, D0632), 1 mM EDTA (Sigma Aldrich, E9884), 0.1% Triton-X 100 (Sigma Aldrich, X100) and EDTA-free protease inhibitors (Roche, 04693116001). Cells were lysed by sonication and centrifuged for 40 min at 20,000 rpm at 4 °C. Samples of all pellet and supernatant fractions were retained at each step of the protocol. The soluble fraction (supernatant) was centrifuged for 1 h at 100,000 rpm at 4 °C and the resultant supernatant was named soluble fraction 1 and the pellet named insoluble pellet 1. Insoluble pellet 1 was passed through a 24-gauge needle to emulsify the sample, then incubated with 1% Triton-X 100 for 1 h at 4 °C with agitation. The sample was centrifuged for another hour at 100,000 rpm at 4 °C resulting in soluble fraction 2 and emulsified pellet 1before repetition of the emulsification/solubilisation step on emulsified pellet 1. One further centrifugation step allowed extraction of soluble fraction 3 and emulsified pellet 2. Each pellet and supernatant sample was assayed for KMO enzymatic activity to determine the location of the protein at each stage of the protocol.

### Solubilisation with detergent

To improve the solubility of cell lysate preparations, various detergents were used as follows.

#### Full length KMO-6His

Bacterial cells were thawed, re-suspended and lysed as before. The crude lysate was incubated with detergents DDM (Sigma Aldrich, D4641), C_12_E_8_ (Sigma Aldrich, P8925), Triton-X 100, Tween20 (Sigma Aldrich, P1379), CHAPS (Sigma Aldrich, P9426) and sarkosyl (Sigma Aldrich, L9150) separately at a final concentration of 1% and incubated for up to 4 h at 4 °C with agitation. Lysates were then centrifuged at 20 000 rpm for 40 min at 4 °C. Denaturing treatment was also carried out using 6 M guanidine hydrochloride (GuHCI), 50 mM DTT and separately, 10 M urea, 50 mM DTT under the same conditions as the detergent screen. Pellet and supernatant fractions were run out on an SDS–PAGE protein gel (Invitrogen, NP0322BOX) with simplyblue staining and analysed by Western blot to detect the protein and determine its solubility.

### Protein purification

#### Truncate KMO

The bacterial cell pellet was thawed and re-suspended in 20 mM HEPES pH7, 200 mM NaCl (Sigma Aldrich, S9888), 10 μM FAD (Sigma Aldrich, F6625) with EDTA-free protease inhibitors. Cells were lysed by sonication and centrifuged to pellet cell debris as above. The soluble supernatant fraction was purified on a 5 ml HisTrap ff crude column (GE Healthcare, 17-5286-01) using the AKTA protein purification system (GE Healthcare). The column was washed with ten column volumes of 20 mM HEPES pH7, 200 mM NaCl and 25 mM imidazole (Sigma Aldrich, I5513). Bound protein was eluted from the column using 20 mM HEPES pH7, 200 mM NaCl and 1 M imidazole. The fractions corresponding to each peak in the chromatogram were combined and dialysed overnight against buffer containing 20 mM HEPES pH7 and 10 μM FAD. The dialysed sample was analysed by SDS–PAGE to determine purity and Western blot analysis was used to identify the truncated KMO protein.

#### Full length KMO-12His-FLAG

The bacterial cell pellet was thawed and re-suspended in 20 mM HEPES pH7, 10 μM FAD with EDTA-free protease inhibitors. Cells were lysed by sonication and centrifuged to pellet cell debris. Protein A magnetic beads (Millipore, LSKMAGA02) were conjugated to anti-FLAG M2 antibody (Sigma Aldrich) by incubation at room temperature for forty minutes with agitation. Beads were then washed with phosphate-buffered saline (PBS) + 0.1% Tween20 before incubating with the soluble supernatant fraction for 2 h at 4 °C with shaking. The bead-antibody-protein complexes were then isolated using a magnet and the beads washed twice before re-suspending in 20 mM HEPES pH7, 10 μM FAD with TEV protease (Sigma Aldrich, T4455) at a ratio of 1:100 of target protein for 4 h at 4 °C. After removal of the beads by magnet, the supernatant was injected onto a GSTrap HP 1 ml column (GE Healthcare, 17-5281-01) to remove GST-tagged TEV protease from the sample. The flowthrough sample containing the KMO-12His protein was dialysed overnight against 20 mM HEPES pH7, 10 μM FAD buffer. The dialysed sample was analysed by SDS–PAGE to determine purity and Western blot analysis was used to identify KMO-12His protein (anti-His(C-term)-HRP Ab at 1:2000 dilution).

### Protein identification

Purified samples were separated using SDS–PAGE and stained with simplyblue before the desired bands were excised from the gel and sent to the Scottish instrumentation and resource centre for advanced mass spectrometry (SIRCAMS) Facility at the University of Edinburgh for identification by mass peptide fingerprinting. The bands were digested by in-gel tryptic digest and the proteins separated by reverse phase chromatography. Peptide fragmentation data was matched against a protein database using the MASCOT search engine.

### Real time-PCR

Full length KMO-FLAG was expressed in BL21 (DE3) pLysS competent cells as before. Non-transformed wild type cells were used as a control. Total RNA was extracted from cell pellets using an RNeasy Mini kit (Qiagen, 74104). 1 μg of total RNA was used for first strand cDNA synthesis using a QuantiTect Reverse Transcription Kit (Qiagen, 205310). Expression of the tryptophan synthase subunit beta (TSSB) gene was determined by real-time PCR. The PCR primers were designed according to published *E*. *coli* ‘BL21 (DE3) pLysS’ genome sequences (GenBank: CP001665.1). Amplification of cDNA samples was carried out using power SYBR green PCR master mix (AB Applied Biosystem, 4309155) under the following conditions: 10 min denaturation at 95 °C, 45 cycles of 15 s at 95 °C and 1 min at 60 °C. This was followed by product melting to confirm single PCR products. Thermal cycling and fluorescence detection were conducted in a StepOne real time PCR system (AB Applied Biosystem). All reactions were carried out in triplicate and the cycle threshold (Ct) number of the targeted gene in each sample was obtained.

## Results

### Enzyme activity of full-length KMO-6His is restricted to the insoluble fraction

**Fig. 3:**
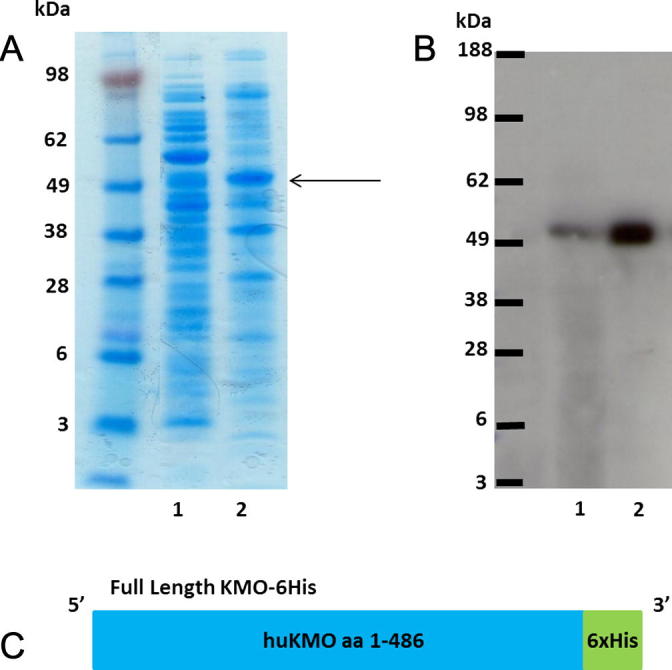
Insoluble flKMO. (A) SDS-PAGE protein gel stained with simplyblue. Lane 1. Soluble fraction following over-expression of full length KMO in bacterial cells. Lane 2. Insoluble fraction, KMO protein (55 kDa) highlighted by arrow. (B) Western blot of the same samples, chemiluminescence detection using Anti-His(c-term)-HRP antibody. Lanes as before. The majority of the KMO protein is present in the insoluble fraction. (C) Cartoon illustrating the flKMO-6His construct. (For interpretation of the references to colour in this figure legend, the reader is referred to the web version of this article.)

Expression of the full length KMO protein was apparent in the insoluble fraction as shown by the 55 kDa band on the SDS–PAGE gel (Fig. 3A). KMO was found to account for 15.5% of the total protein in the insoluble fraction as estimated by gel analysis using ImageJ software. Following Western blot analysis of soluble and insoluble fractions, a comparatively small quantity of protein was found to be soluble (Fig. 3B). Attempts to solubilise the enzyme using various detergents and denaturing agents were not successful. In a kinetic assay measuring KMO activity in each fraction by the production of 3HK, KMO activity correlated with protein expression levels in soluble and insoluble fractions, with significant KMO enzymatic activity demonstrated in the insoluble pellet fraction. This activity remained in the pellet fraction despite several emulsification and centrifugation steps indicating that KMO protein is membrane/protein associated (Table 1).

The small quantities of protein found in the soluble fraction did not bind to NiNTA affinity or ion exchange chromatography resin.

### C-terminal truncates of huKMO are soluble but enzyme activity is lost

**Fig. 4:**
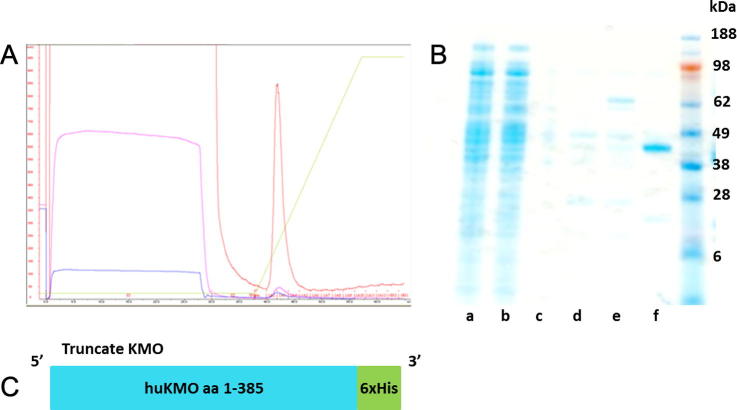
Purified trKMO. (A) Chromatogram produced during NiNTA affinity purification of truncate KMO. (B) SDS-PAGE protein gel stained with simplyblue showing HisTrap purification of truncated human KMO protein, (a) Cell-free extract prior to affinity chromatography, (b) flowthrough proteins which did not bind to the column, (c) first column wash, (d) second column wash, (e) third column wash, (f) pure truncated human KMO protein (44 kDa) from the fractions under the chromatogram. (C) Cartoon illustrating the trKMO construct. (For interpretation of the references to colour in this figure legend, the reader is referred to the web version of this article.)

C-terminal truncated KMO protein was readily expressed in the soluble fraction. The soluble enzyme was purified by NiNTA affinity chromatography as indicated in Fig. 4A. However, soluble truncated KMO protein did not demonstrate enzymatic activity in a crude bacterial cell lysate or purified form as indicated by no production of 3-HK. The identity of the purified 44 kDa protein band was determined by mass peptide fingerprinting to be human kynurenine 3-monooxygenase (Table 2).

### Full length huKMO-12His-FLAG is soluble and active

**Fig. 5:**
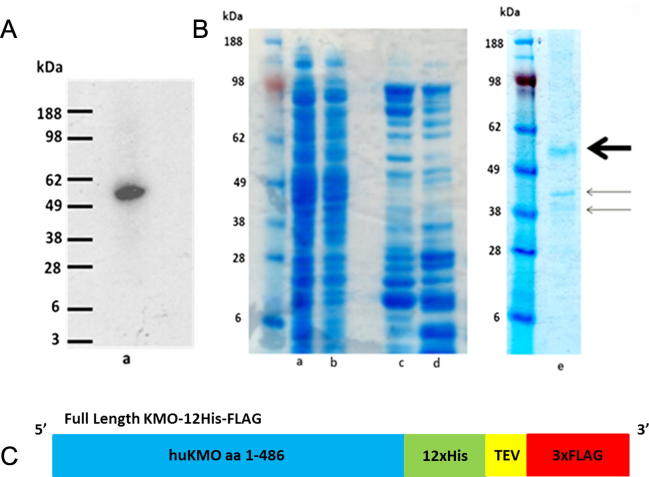
Soluble partially purified KMO-FLAG. (A) Western blot using chemiluminescence detection, anti-FLAG M2 antibody and goat anti-mouse HRP secondary antibody, (a) soluble fraction from over-expressed KMO-12His-FLAG (57 kDa) in bacterial cells. (B) SDS-PAGE gel stained with simplyblue showing anti-FLAG conjugated protein A magnetic bead purification of KMO-12His-FLAG, (a) Cell-free extract prior to purification, (b) flowthrough proteins which did not bind to the beads, (c) first magnetic bead wash, (d) second bead wash, (e) purified KMO-12His (57 kDa) (bold arrow) eluted from the beads by TEV protease has precipitated with two contaminant *E*. *coli* proteins (indicated using arrows). (C) Cartoon illustrating the flKMO-12His-FLAG construct. (For interpretation of the references to colour in this figure legend, the reader is referred to the web version of this article.)

Expression of soluble FLAG tagged KMO was indicated by a 57 kDa band on an SDS-PAGE gel and validated by Western blot (Fig. 5A). Enzymatic activity of the protein expressed in the soluble fraction was indicated by significant 3-HK production.

#### Purification

Partial purification was achieved using affinity chromatography on anti-FLAG M2 antibody conjugated protein A magnetic beads (Fig. 5B). KMO was found to account for 71% of the total protein remaining after purification as determined by gel analysis using ImageJ software. This partially purified fraction contained 1.8 mg total protein meaning that ∼19% of recombinant KMO was recovered from the soluble fraction. The SDS-PAGE gel indicates that purified KMO protein (57 kDa) co-purifies with two other protein bands. These proteins were identified as *E*. *coli* proteins tryptophan synthase beta subunit and a putative GTP binding protein by mass peptide fingerprinting (Table 3). Real Time-PCR results showed no significant difference in expression of tryptophan synthase beta subunit mRNA between BL21(DE3) pLysS cells expressing KMO-FLAG and wild type control cells. The RQ value for the control cells was set at one for comparative purposes and the measured RQ value for pLysS cells expressing KMO-FLAG was 0.72.

#### Kinetic characterisation

**Fig. 6:**
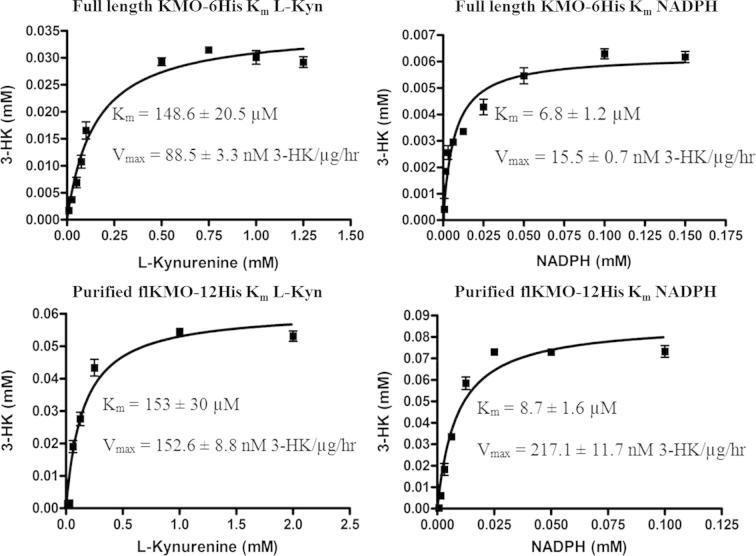
Kinetic characterisation of KMO. Steady state kinetics for flKMO-6His and purified KMO-12His (FLAG removed during purification) at 37 °C, pH 7. Starting concentrations of kynurenine and NADPH are plotted versus 3-HK produced for each protein and data fitted to the Michaelis–Menten equation (*v* = *V*_max_.[l-Kyn]/(*K_m_*+[l-Kyn]) using GraphPad Prism4 software.

Previous studies have shown that it is possible to monitor NADPH oxidation by absorbance to measure KMO activity. However, this assay is insensitive and requires higher levels of purified protein. Since the level of KMO expression was low we used an LC–MS/MS assay for the accurate determination of steady state kinetic parameters. For all assays turnover of kynurenine in this stopped kinetic assay was in the linear range thus allowing accurate measurement of the initial reaction velocities to fit the *K_m_* for each substrate. Full length KMO-6His demonstrated a *K_m_* for l-Kynurenine of 148.6 ± 20.5 μM and a *K_m_* for NADPH of 6.8 ± 1.2 μM (Fig. 6). Purified full length KMO-12His (FLAG tag removed during affinity purification) was found to have a *K_m_* for l-Kynurenine of 153 ± 30 μM and a *K_m_* for NADPH of 8.7 ± 1.6 μM. Steady state kinetic data were plotted using GraphPad Prism version 4.0 and the data was fitted to the Michaelis–Menten equation (*v* = *V*_max_.[l-Kyn]/(*K_m_* + [l-Kyn]). (Fig. 6). Expression parameters for all three protein constructs are summarised in Table 4.

## Discussion

Human KMO is a drug development target of increasing importance. Here, we report the first successful expression of huKMO in a bacterial expression system and describe the partially purified, FLAG-tagged recombinant human enzyme expressed in *E*. *coli.* Full-length 6× histidine tagged human KMO protein demonstrated significant expression but remained predominantly in the insoluble fraction. The comparatively smaller quantity of protein found in the soluble fraction did not demonstrate affinity for NiNTA or cation exchange chromatography resin despite the 6xhistidine tag and net positive charge. The putative mitochondrial membrane-targeting C-terminal domain may be responsible for protein insolubility [9]. The amount of KMO in the soluble fraction was not improved by membrane protein isolation and detergent solubilisation techniques. It is reasonable to assume that huKMO localises to the pellet fraction at each step of the membrane protein isolation protocol because it is membrane-protein associated *in vivo*. KMO was not successfully extracted from the pellet fraction at any stage, despite exhaustive efforts, indicating formation of very tightly bound aggregates. Membrane or protein association could explain the inaccessibility of the protein for purification by affinity chromatography. Despite the insoluble and aggregated state of this construct, the protein did demonstrate KMO enzymatic activity. As the protein exists on the outer mitochondrial membrane in its native state, a membranous environment may be desirable for the activity of the recombinant enzyme.

In an attempt to improve solubility of the human enzyme, a truncated construct was created which lacked the coding sequence for 100 amino acids at the C-terminus of the expressed protein, thus removing the mitochondrial outer membrane targeting signal. This truncated protein was readily expressed in the soluble fraction, was purified without difficulty and was identified as huKMO by mass peptide fingerprinting. However, this truncated protein did not demonstrate production of 3-HK in the kinetic assay. Concurrent with these experiments, Hirai et al [15] reported that the C-terminus of pig KMO has a dual role in that it is required for enzymatic activity as well as membrane targetting. That group showed that positions −30 to −50 from the C-terminus are necessary for activity of the pig enzyme [15]. Our results corroborate those of Hirai et al [15] as a corresponding C-terminal domain was absent in our truncated human construct. Removal of amino acids 386–486 of the human enzyme resulted in soluble protein but loss of enzymatic activity. This result differs from the recently reported yeast KMO crystal structure where a truncated 1-390 residue yeast KMO was reported to demonstrate enzymatic activity. Whilst alignments (not shown) confirm that the active site of KMO is conserved between yeast and human KMO, homology between the two is fairly poor at approximately 33% identity. The regions remaining in the human truncate reported here are similar when compared to this yeast truncate, however, the human construct is not active. This indicates that a domain which is critical for the retention of enzymatic activity in human KMO is absent or not required for enzymatic activity in the yeast protein. Because extension of a protein by FLAG tag fusion has been shown to increase solubility and stabilize heterologously expressed proteins [18], we adopted this technique. Generation of a FLAG-tagged fusion protein vastly improved the solubility whilst retaining activity. This agrees with, and may be explained by, the observations of Hirai et al [15] using pig KMO in which the C-terminal FLAG tag weakened the positive charge and hydrophobicity required for membrane targetting [15].

The FLAG tag epitopes of the huKMO fusion protein, being maximally hydrophilic [18], were sufficiently exposed to allow affinity purification. NiNTA affinity and anion exchange chromatography techniques were attempted for purification of full Length KMO-12His-FLAG but these purification systems were less successful, possibly due to the inaccessibility of tags or aggregation. The steady state kinetic parameters obtained for each substrate for the purified enzyme were comparable to those reported for the human enzyme when expressed in mammalian cells [8]. Approximately 19% of the bacterially expressed enzyme was recovered during purification. This compares with 60% overall recovery of KMO expressed in mammalian cells [8]. Following purification, huKMO precipitated with two contaminant *E*. *coli* proteins, identified by MALDI-TOF as the beta subunit of tryptophan synthase and a putative GTP-binding protein. Tryptophan synthase catalyses the final two steps in the biosynthesis of tryptophan [19]. The enzyme typically exists as an α–ββ–α complex and a long hydrophobic channel links each α and β active site [20]. The existence of this hydrophobic channel within the enzyme may explain its co-elution and potential interaction with KMO during purification as the two hydrophobic molecules may form complexes of unknown stoichiometry. Because increased KMO activity in bacteria might drive increased expression of upstream enzymes to maintain substrate levels and homeostasis, we tested whether native bacterial TRP-synthase expression was increased. Semi-quantitative RT-PCR confirmed no increase in native TRP-synthase mRNA expression, therefore, the co-elution was more likely to be due to complex formation or binding rather than upregulation of protein expression.

Many GTP-binding proteins are located on or bind to the plasma membrane of cells. The precise identity of the second co-precipitant with KMO was not determined, since mass peptide fingerprinting techniques for protein identification can be limited by differential sensitivity of detection for individual peptides [21]. In the paper of Hirai et al [15], removal of co-eluting cytochrome b5 and monoamine oxidase subunit B abolished KMO enzymatic activity and these proteins were shown to form protein-protein complexes with pig KMO via tight hydrophobic interactions [15]. The bacterial cells used in our system do not express these proteins. Partially-purified huKMO remained active in the absence of these proteins, suggesting firstly that *E*. *coli* provides functional analogues for cytochrome b5 and MAO-B, or maybe that under our conditions cytochrome b5 and MOA-B are not necessary for huKMO activity. It is plausible that interaction of KMO with tryptophan synthase and the GTP-binding protein may be creating a hydrophobic environment for KMO, thus permitting activity to be retained after partial purification and removal of the membranous environment in which KMO activity is optimal.

This is the first report of successful expression of active human KMO enzyme in a bacterial expression system with specific fusion protein constructs allowing partial purification and further characterisation of the human KMO enzyme. These insights together with the newly reported yeast KMO structural information provide useful information for future studies on this important therapeutic target enzyme. However, it will be necessary to achieve improved yields of the human enzyme to enable large scale *in vitro* screening of potential KMO inhibitors and for the elucidation of the human KMO crystal structure.

## Funding Information

This work was supported by the Medical Research Council.

## Figures and Tables

**Table 1:** Membrane associated KMO. Membrane protein isolation protocol samples show that KMO activity is significantly higher in the insoluble pellet fractions indicating that KMO is membrane/protein associated.

Sample	Total protein assayed (μg)	3-HK produced (μM)
Soluble fraction 1	200	1.4
Soluble fraction 2	200	7.6
Insoluble pellet 1	200	79.7
Emulsified pellet 1	200	68.7
Soluble fraction 3	200	None detected
Emulsified pellet 2	200	160.2

**Table 2:** Positive KMO ID. Mass peptide fingerprinting results identified the purified protein gel band as KMO. A probability based Mowse score above 50 is acceptable as identity.

Protein ID	Mowse score
Human kynurenine 3-monooxygenase	535

**Table 3:** Identification of co-eluting proteins. KMO-FLAG contaminant *E*. *coli* proteins identified by mass peptide fingerprinting and their corresponding Mwt and Mowse scores.

Protein ID	Molecular weight (Da)	Mowse score
Tryptophan synthase, beta subunit	43 670	437
Putative GTP-binding protein	39 930	340

**Table 4:** Summary of protein expression. Constructs used for bacterial expression of KMO exhibited a variety of expression parameters.

Protein construct	Expressed	Soluble	Active	Yield
Full length KMO-6His	Yes	No	Yes	nd
Truncate KMO	Yes	Yes	No	nd
Full length KMO-12His-FLAG	Yes	Yes	Yes	19%

nd, not determined.

## Footnotes

1 *Abbreviations used:* KMO, Kynurenine 3-monooxygenase; NAD, nicotinamide adenine dinucleotide; 3-HK, 3-hydroxykynurenine; FAD, flavin adenine dinucleotide; trKMO, Truncated KMO; flKMO, Full length KMO-6His; KMO-FLAG, Full length KMO-12His-FLAG; PFP, pentafluorophenyl; GuHCI, guanidine hydrochloride; PBS, phosphate-buffered saline; SIRCAMS, Scottish instrumentation and resource centre for advanced mass spectrometry; TSSB, tryptophan synthase subunit beta.
